# Investigation of Bacterial Cellulose Biosynthesis Mechanism in *Gluconoacetobacter hansenii*

**DOI:** 10.1155/2014/836083

**Published:** 2014-03-16

**Authors:** Bhavna V. Mohite, Satish V. Patil

**Affiliations:** School of Life Sciences, North Maharashtra University, P.O. Box 80, Jalgaon 425001, India

## Abstract

The present study explores the mechanism of cellulose biosynthesis in *Gluconoacetobacter hansenii*. The cellulose synthase enzyme was purified as membrane fraction and solubilized by treatment with 0.1% digitonin. The enzyme was separated by native-gel electrophoresis and **β**-D-glucan analysis was carried out using in vitro gel assay. The cellulose synthase has glycoprotein nature and composed two polypeptide subunits of 93 KDa and 85 KDa. The confirmation of **β**-1,4-glucan (cellulose) was performed in whole and hydrolyzed monomeric sugar form. Tinopal and Congo red were used for cellulose detection on the gel. Thus the in vitro cellulose synthesis assay with cell free enzyme fraction was attempted to improve the understanding of cellulose biosynthesis.

## 1. Introduction

Cellulose is one of the most abundant macromolecule on earth. In spite of the importance of cellulose, its mechanism of biosynthesis is poorly understood.* Acetobacter xylinum* become a model system to study the synthesis of cellulose [1]. The first major advance in the area of (1-4)-*β*-glucan synthesis came from a prokaryotic organism,* Acetobacter xylinum* [2]. The cellulose synthase from this organism uses uridine diphosphate-glucose (UDP-glucose) directly as a substrate for polymerization in vivo and in vitro [2].

In the past decade, a number of new developments in the biological and cytological aspects of cellulose biosynthesis from Acetobacter have led to better understanding of this process. Cellulose biosynthesis is an exciting area of study with lots of challenges and opportunities [3]. The enzymatic pathway for cellulose synthesis in* A. xylinum* has been extensively investigated and four essential enzymatic steps have been identified [4]. Cellulose synthase is the only enzyme known to be unique to the cellulose synthetic pathway. Biosynthesis of cellulose essentially proceeds by polymerization of glucose residues using an activated substrate (UDP-glucose). In* Acetobacter xylinum*, the enzyme cellulose synthase is present on the cytoplasmic membrane, and the cellulose product is obtained extracellularly [5].

Successful purification of cellulose synthase and being acquainted with its properties would augment our understanding of molecular mechanism of cellulose synthesis [6]. Partially purified membrane protein was used for assaying cellulose synthase activity. UDP-glucose is the direct substrate, from which glycosyl residues transfer to the nonreducing end of the growing (1→4)-*β*-glucan chain. The formation of cellulose, that is, the polymerization of glucose, appears to be catalyzed by the gene product of the* bcsA* gene product of the* bcs *(bacterial cellulose synthase operon) [7]. There is a coupling of cellulose synthesis and translocation in which the nascent polysaccharide is extended by one glucose molecule at a time [8].

In the present study, in vitro *β*-1,4-glucan synthesis was directed towards understanding the mechanism of cellulose biosynthesis. Later, conformation of formed cellulose was detected with its intact form and after hydrolysis into monomer sugar. In addition, we illustrated the electrophoretic separation of cellulose synthase and determination of its mass and glycoprotein nature.

## 2. Materials and Methods

### 2.1. Microorganism

*Gluconoacetobacter hansenii* NCIM 2529 was grown in Luria Bertani (LB) broth (Himedia, Mumbai, India) on shaker at 28°C overnight. The cell pellet was separated by centrifugation at 18,000 g for 20 min at 4°C.

### 2.2. Preparation of Membrane Fractions: Solubilization of Enzyme

The cell pellet was suspended in 50 mM Tris-HCL, pH 7.5 containing 5 mM EDTA, followed by a brief sonication and recentrifugation at 10,000 ×g for 5 min at 4°C to remove cell wall. Membrane pellet was collected and used as source of enzyme.

Enzyme was solubilized by resuspending the membranes in 0.05 M Tris-HCL, pH 7.5, containing 22 mM MgCl_2_, 1 mM EDTA, and 0.1% (w/v) digitonin. The suspension was placed in a sonic bath for 5 min at 1–4°C and then stirred on ice for 30 min followed by centrifugation at 100,000 ×g for 1 h. The supernatant represented the solubilized enzyme [6].

### 2.3. Native Polyacrylamide Gel Electrophoresis

The digitonin supernatant was applied directly as enzyme sample; after application of samples (5–10 *μ*L each containing 10–50 *μ*g protein), electrophoresis was carried out using the discontinuous buffer system without the addition of SDS. A Bangalore Genei vertical minislab gel apparatus was used; the small size of the gels (8 cm × 7 cm) reduces the volume of solution required in the activity assay and enhances diffusion of substances (e.g., substrate) between gel and solution. Stacking gel (4.5%) and separating gel (10%) acrylamide were used. Native-gel electrophoresis was performed at 4°C, at 10 to 15 mAmp per gel. The 50 v current was applied initially and then increased to 100 v after the electrophoresis run reached up to the separating gel. The position of bands for molecular weight was determined by comparison with molecular weight markers (high range molecular weight markers 29 to 250 KD cat# 105977 Merck inst.). Among the bands obtained, a band of cellulose synthase complex (predicted molecular mass ~175 KDa) was selected according to previous report [9] and it was further purified by using gel extraction kit (Banglore Genei).

### 2.4. *β*-D-Glucan Product (Cellulose) Assay in Native Gel

Following electrophoresis, gel lane was cut into sections containing sample lane and rinsed in 100 mL of 10 mM Tris-HCL (pH 7.5) for 30 min and buffer was changed after 15 min. Each gel section was then placed in a plastic petri dish and incubated for enzyme activity at room temperature in 50 mM Tris-HCL (pH 7.5), 3 mM NaN_3_, and UDP-glucose (Sisco Research Laboratories, India). Unreacted substrate was removed from gel sections by rinsing twice for 20 min each in 100 mL of 5 mM EDTA, 10 mM Tris-HCL (pH 7.5). The *β*-glucan product in the gel was visualized with Congo red after staining in 50 mL of 0.1% Congo red for 30 min and destaining for 20 min in 100 mL of 10 mM Tris-HCL (pH 7.5) buffer.

An alternative method was employed to detect the *β*-glucan product. After reaction with the substrate UDP-glucose, the *β*-glucan product was visualized by its fluorescence under UV light staining with Tinopal (4,4′-distyrylbiphenyl sodium sulfonate salt, Sigma-Aldrich) in solution of 50 mL of 0.01% Tinopal blue in K_2_PO_4_ buffer, pH 8.2, for 10 min. The gel was then washed in water and stored in the same solution.

### 2.5. Analysis of *β*-Glucan Product (Cellulose)

The product (cellulose) formation was confirmed by two major ways: (i) detection of cellulose and (ii) detection of cellulose degradation product (monomer sugar).

*(i) Detection of Cellulose*. The formation of reaction product (cellulose) in cellulose synthase assay was detected by following ways. (a) The thin layer chromatogram of UDP-glucose and reaction product was developed with methanol as solvent system. TLC was visualized under short UV for fluorescence. The UV-visible spectrum of UDP-glucose and reaction product was also carried out by Nanodrop spectrophotometer (ND 1000, Nanodrop technology, USA). (b) The second one is reaction with Congo red. (i) The reaction product (cellulose) and substrate (UDP-glucose) as control were streaked on silica gel plate and the plate was sprayed with Congo red solution to observe the characteristic binding of Congo red. (ii) The reaction product was mixed with Congo red solution and centrifuged for 10 min at 5000 g. The colour of precipitated cellulose at bottom was observed. Binding of Congo red was also confirmed by measuring the spectrum by UV-visible Nanodrop spectrophotometer.

*(ii) Detection of Cellulose Degradation Product (Monomer Sugar). *The degradation product of cellulose, that is, monomer glucose sugar, was detected for the confirmation of cellulose as reaction product in cellulose synthase assay. The reaction product was precipitated with isopropyl alcohol and used for further assays.

The reaction product was used as substrate for cellulase assay. Reaction mixture precipitate was treated with cellulase enzyme (Himedia, Mumbai, India) and allows it to react for 60 min at 50°C in a water bath for the hydrolysis. After hydrolysis of reaction product with cellulase enzyme, reducing sugar was measured by Millers method [10]. The hydrolysed product of cellulose was confirmed as glucose by thin layer chromatogram (TLC) and Fourier transform infrared chromatography (FTIR). The hydrolysis product of cellulose assay was spotted on TLC (Silica gel Hi-250 F) plate with glucose as standard and chromatogram was run with chloroform: glacial acetic acid: water (30 : 35 : 5) as solvent system. Detection was carried with chromogenic iodine (CIR) as spraying reagent. The FT-IR transmission spectrum of the cellulase enzyme treated product was studied to prove the purity of the reaction product (1,4-*β*-D-glucan), made up of only glucose monomer compared with glucose as standard.

### 2.6. SDS PAGE Electrophoresis of Purified Enzyme Complex

The protein band from native PAGE was further characterized by sodium dodecyl sulphate-polyscrylamide gel electrophoresis (SDS-PAGE) with 10% SDS. The sample was dissolved in sample buffer containing 10% SDS, 0.2 M Tris-HCL (pH 6.8), 10 mM-mercaptoethanol, and 20% glycerol with 0.05% bromophenol blue. The incubation was carried out on ice for 1 h to avoid smearing protein bands due to heating. Gel was stained overnight with 0.125% Coomassie brilliant blue R-250 prepared in 40% methanol and 10% acetic acid and destained for 2 h with the same lacking Coomassie blue. The position of bands for molecular weight was determined by comparison with molecular weight markers (high range molecular weight markers 29 to 250 KD cat# 105977 Merck inst.).

### 2.7. Glycoprotein Identification

The native gel electrophoresis was run and a lane was cut and incubated in the fixing solution (25% isopropanol, 10% acetic acid, and 65% water) for 2 h with gentle shaking. The fixation was repeated for another hour with change of fixing solution. Then gel was transferred into equilibrium solution (0.2% thymol blue (w/v) in fixing solution) and incubated for 2 h. Then equilibrium solution was totally decanted and staining solution (80% sulphuric acid 20% ethanol) was added and incubated the gel for 3 h. Reddish brown colour bands of corresponding glycoprotein lighten up and are documented by photography.

## 3. Results and Discussion

Many different approaches have been studied to investigate synthesis of cellulose [11, 12]. Significant efforts have been made to achieve in vitro synthesis of cellulose with membrane preparations of various degrees of purity from different organisms [13–15]. In the present study, we demonstrated the mechanism behind cellulose synthesis and polymerization in* G. hansenii* NCIM 2529. The role of cellulose synthase was detected and characterized using an* in situ *assay following solubilization and electrophoretic separation in nondenaturing polyacrylamide gels. The enzyme which is extraordinarily unstable in extracts at ambient temperature maintains at least some activity in the gel assay. Therefore native gel assay was used rather than solution assay.

### 3.1. Determination of Cellulose Synthase Activity

When cellulose biogenesis was considered as a universal phenomenon, much interest was obviously focused upon the cellulose synthase, since this may well be the only enzyme unique to this process [16]. The cellulose synthase is most probably an integral membrane protein that occurs exclusively in the membrane-associated fraction, as determined for a variety of* A. xylinum* strains and* Agrobacterium tumefaciens *[17]. Successful separation of cellulose synthase and its activity depends on efficient membrane solubilization and extraction of active proteins [18]. Digitonin was reported as the best for solubilization activity for Acetobacter cellulose synthase [19] compared with Triton, Zwittergent, and cholate and hence it was used in this study. Brief sonication and extraction of Acetobacter membranes with buffer containing digitonin (1–10%) result in effective solubilization of the UDP-glucose: l,4-P-D-glucan 4-P-D-glucosyltransferase (cellulose synthase) with very good recovery of activity [19]. After electrophoretic separation of proteins in the membrane fractions by nondenaturing polyacrylamide gel electrophoresis (PAGE), the product of *β*-glucan synthase activity can be demonstrated by the cellulose synthase enzyme assay by providing UDP-glucose as a substrate and cofactors. The in vitro assay system includes synthesis of alkali insoluble, *β*-1,4-glucan (cellulose), from UDP-glucose which is added as the sole, exogenous substrate, at rate comparable (40%) to that of the whole cell [20]. UDP-glucose has been characterized as glucosyl donor for cellulose synthesis [21]. According to Delmer et al. [22] in vitro rate of cellulose synthase is half of that rate observed in vivo. The in vitro synthesis of cellulose was confirmed by (a) detecting the cellulose formed and (b) detecting the hydrolysis product of cellulose, that is, monomer sugar, glucose.

### 3.2. Detection of Cellulose Formation

**Figure 1:**
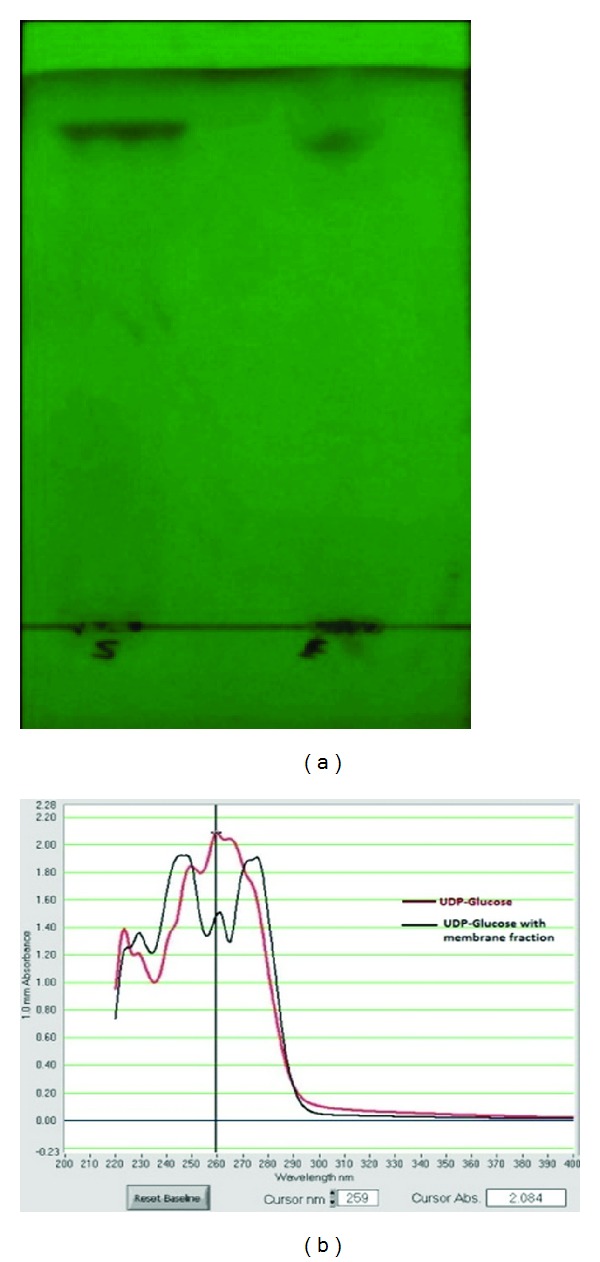
(a) Thin layer chromatogram showing UV fluorescence by UDP-glucose; (b) UV-Vis spectrum of UDP-glucose and reaction product.

*(a) The UDP-Glucose Gives Fluorescence Compared with the Reaction Product (Cellulose) (Figure 1(a)). *The UDP-glucose when reacting with cellulose synthase from the membrane fraction results into cellulose which did not fluoresce under UV light; this gives a primary indication of conversion of UDP-glucose into cellulose. The UDP-glucose and its transformation into product were confirmed by observing the decrease in concentration of UDP-glucose after reaction with membrane fraction enzyme (Figure 1(b)).

**Figure 2:**
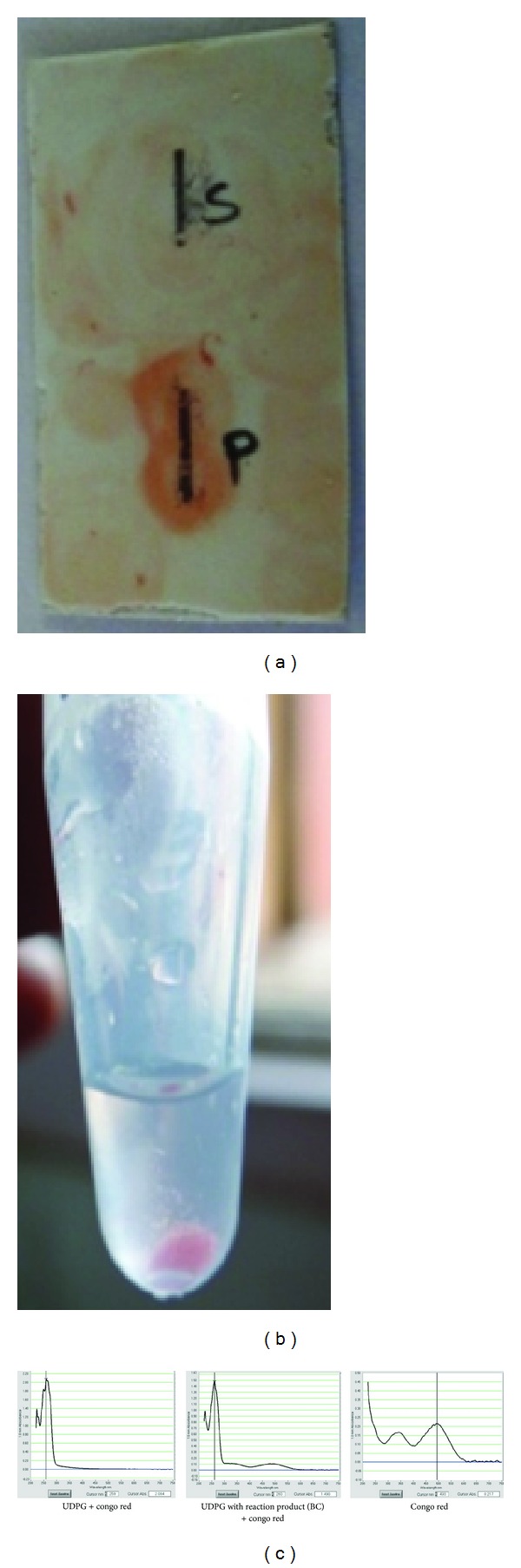
(a) Characteristic binding of Congo red with reaction product. (b) Precipitate of red colored cellulose. (c) Spectrum of UDPG and reaction product with Congo red compared with spectrum of Congo red.

*(b) Detection with Congo Red*. Binding of dye with cellulose: (i) when the reaction mixture (product) was streaked on TLC plate along with UDP-glucose as substrate control and sprayed with Congo red solution, red colour was developed only with the reaction product when compared to control (Figure 2(a)). This happens due to characteristic binding affinity between cellulose and Congo red. (ii) The precipitated red coloured cellulose (product) was observed due to characteristic binding of Congo red to cellulose (Figure 2(b)). As the polymerization further proceeded, the precipitate of the reaction product (polysaccharide, cellulose) appeared in the solution. This indicated the self-assembly of cellulose (water insoluble) synthesized by in vitro enzymatic polymerization [23]. (iii) The precipitated reaction product and UDP-glucose with Congo red were compared spectrophotometrically which indicate that Congo red shows binding with the reaction product, that is, cellulose not with UDP-glucose (Figure 2(c)). This confirmed formation of cellulose as reaction product by catalysis with cellulose synthase enzyme separated from membrane fraction. UDPG and UDPG + enzyme fraction (= BC) reacted with Congo red and had been measured on Nanodrop spectrophotometer (Figure 2(c)).

**Figure 3:**
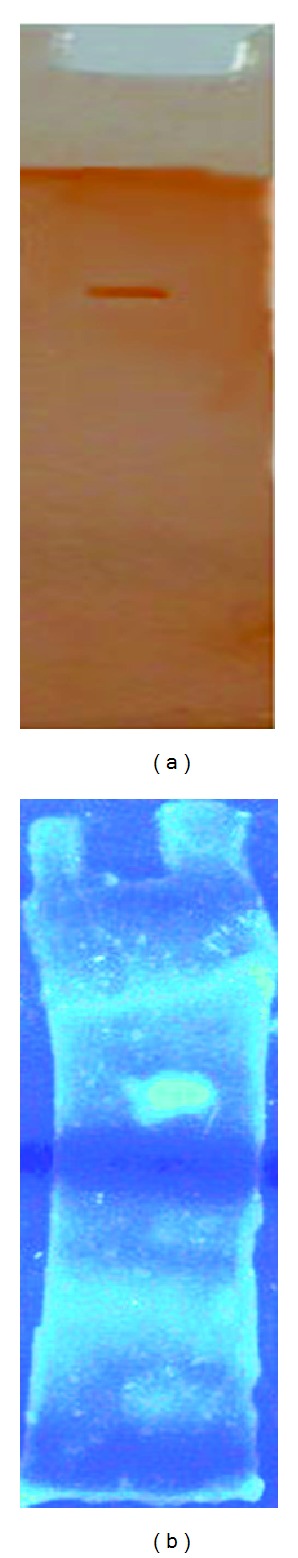
Detection of cellulose on native gel by (a) Congo red and (b) Tinopal CBS under UV light.

*(c) Detection of Cellulose Synthase Product (Cellulose) in the Native Gel.* Following separation on nondenaturing gel, the activity of cellulose synthase can be analyzed by incubating washed gel with appropriate substrate and effector, subsequent removal of unused substrate by washing, followed by the use of suitable detection procedure. The gel assay is highly sensitive than solution assay as the product formed was detected on the gel itself without loss of product [6]. Figures 3(a) and 3(b) demonstrate such an assay for detection of formed cellulose by binding with Congo red and Tinopal CBS, respectively. The Congo red reacts with cellulose and gives red coloured band on gel. Tinopal reacted with cellulose and was detected under UV with fluorescence due to binding with optical brightener, Tinopal. The insolubility of produced *β*-glucan helps to immobilize in the gel matrix, thus suggesting that this product was *β*-1,4-glucan.

*Detection by Analyzing the Degradation Product (Monomer Sugar) of *β*-Glucan Product (Cellulose). *The formation of reaction product as cellulose was also confirmed by detecting the degradation product of cellulose, that is, monomeric form of sugar (glucose). The cellulose was precipitated with isopropyl alcohol and hydrolyzed with cellulase enzyme by cellulase assay. The detection of glucose as a result of hydrolysis of cellulose confirms the purity of cellulose formed [24].

*Cellulase Assay*. The cellulase assay was performed with the product of in vitro assay as substrate (cellulose). In cellulase hydrolysis assay, glucose liberated from cellulose was quantified by Millers method with dinitrosalicylic acid which specifically reacts with the free sugar. The concentration of reducing sugar after cellulase hydrolysis was much higher (115 *μ*g/mL) compared with unhydrolyzed reaction product [25]. The positive cellulase assay confirmed that the substrate for cellulase assay was of polymeric **β**-1,4-glucan nature.

The hydrolysis product of cellulose (i.e. glucose) was detected by thin layer chromatography (TLC) and Fourier transform infra red spectroscopy (FTIR). The hydrolysis of cellulose with cellulase results into release of glucose as monomer sugar.

**Figure 4:**
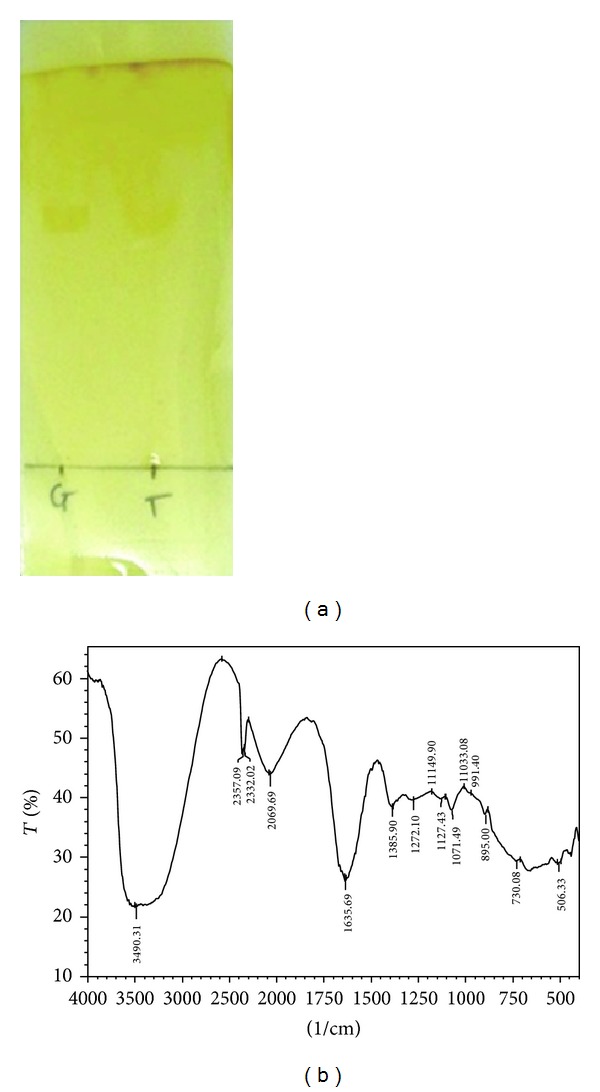
(a) Thin layer chromatogram of cellulase hydrolyzed reaction product (T) compared with glucose (G) as standard. (b) FT-IR spectrum of cellulase treatment reaction product.

- In TLC, the Rf value was corresponding with standard glucose that confirmed that the reaction product under cellulase assay was cellulose (Rf value of test- 0.63, matching with Rf value of glucose- 0.61) (Figure 4(a)).
- The cellulase treated reaction product was matched with glucose based on FT-IR spectrum (Figure 4(b)). FTIR spectrum of aqueous solution of cellulase enzyme hydrolysate was well defined and shows intense and characteristic bands in the region between 1200 and 900 cm^−1^. The characteristic bands of glucose have specific maxima at 991, 1033, 1078, 1107, and 1149 cm^−1^, with the peak at 1033 cm^−1^ having the highest absorption which is a characteristic to the C–O stretch vibration. Glucose has endocyclic C–O located at around 1080 cm^−1^. The peak at 1033 cm^−1^ was considered for the identification of cellulase hydrolytic activity and the rate of glucose release [26, 27].

### 3.3. SDS PAGE of Purified Cellulose Synthase

**Figure 5:**
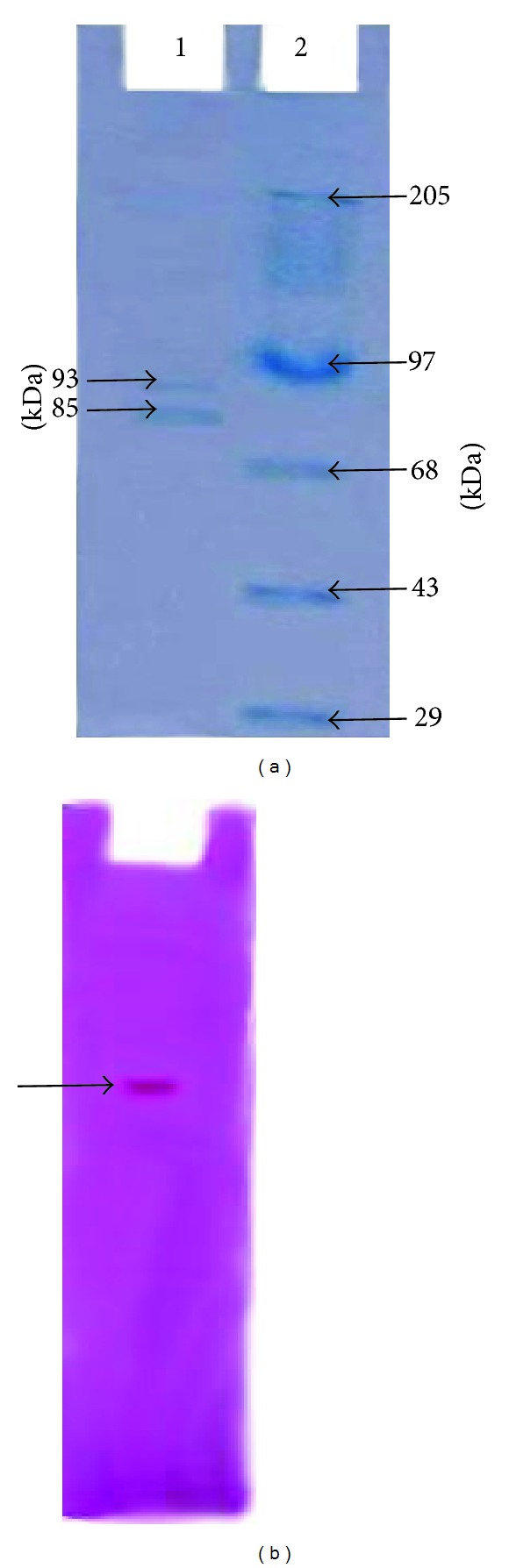
(a) SDS-PAGE of cellulose synthase (lane 1) purified from* G. hansenii* stained by Coomassie brilliant blue compared with high range molecular weight marker (lane 2). (b) Glycoprotein band of cellulose synthase on native gel.

The bands detected by Coomassie brilliant blue staining was compared with high range molecular weight marker. Two distinct bands were observed corresponding to molecular weight of 93 KDa and 85 KDa (Figure 5(a) lane 1) compared to high range molecular weight marker showing bands of 205, 97. 68, 43, and 29 KDa (Figure 5(a) lane 2). This result strongly implicated the involvement of two subunit polypeptide with cellulose synthase [28] and this work as catalytic subunit of cellulose synthase [29]. Multiple catalytic subunits are required for cellulose synthesis in* Arabidopsis* [30, 31].

### 3.4. Glycosylated Nature of Cellulose Synthase Enzyme Complex

The cellulose synthase enzyme on a gel was stained with thymol blue for determination of its glycoprotein nature. The glycoprotein nature of the protein is detected by staining method where amino group of amino acid and aldehyde or keto group of the sugar in the protein leads to the reddish brown colour of the band. Glycoprotein band was visualized in reddish brown colour which confirmed the glycoprotein nature of cellulose synthase enzyme (Figure 5(b)).

## 4. Conclusion

The present study attempted to reveal the mechanism behind bacterial cellulose production and polymerization. Extraction and purification of membrane protein were carried out with its elctrophoretic separation. The two subunit polypeptides of 93 KDa and 85 KDa with glycoprotein nature implicated cellulose synthase. The in vitro *β*-1,4-glucan gel assay was carried out to determine role of cellulose synthase and further *β*-glucan product was analyzed. Formation of *β*-glucan product was confirmed in whole and hydrolyzed sugar monomer. Thus the mechanism of bacterial cellulose formation was elucidated with involvement of cellulose synthase that would facilitate understanding of cellulose synthesis which could be employed for more productivity of cellulose from cellulose producing strains.
